# Materia Medica and Therapeutics; Medical Chemistry; Toxicology and Legal Medicine

**Published:** 1860-05

**Authors:** Emil Fischer


### MATERIA MEDICA AND THERAPEUTICS; MEDICAL CHEMISTRY; TOXICOLOGY AND LEGAL MEDICINE.

BY EMIL FISCHER, M.D.

### Materia Medica and Therapeutics.

I.—*Medicinal Properties of Globularia Alypum, L.* By Dr. G. Planchon, of Montpellier.

The purgative properties of the leaves of this plant have lately been brought before the medical profession by Dr. Gustave Planchon, of Montpellier.

Dr. Planchon, in an essay entitled *Des Globulaires au point de vue Botanique et Médical*, Montpellier, 1859, discusses, in the first place, the botanical affinities of the genus *Globularia*, which, after careful revision, he concurs with Dr. Lindley in placing in the natural order *Selaginaceæ*. The second part of his pamphlet relates entirely to one species of *Globularia*, namely, *G. Alypum, L.*, a small shrub of the South of Europe, well known to the botanists and physicians of the sixteenth and seventeenth centuries, who generally regarded it as a drastic purgative. Dr. Planchon shows that these ideas of the powerfully cathartic properties of the *Globularia* are very much exaggerated, and that in fact its leaves are a safe, mild, and efficient purgative. The form in which the medicine is best exhibited is a decoction, made by boiling an ounce of the leaves in water for ten minutes. This quantity constitutes one dose. Dr. Planchon has arranged his observations on twenty-two cases in a tabular form, from which we gather that the decoction administered in the dose named, usually acts in about two hours, producing on an average four evacuations. Neither vomiting nor nausea is felt after a dose, nor does its action produce griping. In fact, it purges very gently, without occasioning any unpleasant symptoms. Another advantage which it possesses, and which Dr. Planchon very justly points out as remarkable, is that of not occasioning any subsequent constipation—a fact noticed in every case recorded. It may be employed, says he, in all cases in which a purgative effect is required without irritation of the intestinal canal. In its bitter properties it approaches that class of purgatives which, taken in small doses, exercise on the digestive organs a simply tonic action. It fulfills the same indications as rhubarb, but with the advantage of purging more mildly and of not producing the ulterior constipation, which is one of the commonest inconveniences of that medicine.—(*Pharmaceutical Journal and Transactions*, London, February, 1860.)

II.—*Pharmaceutical Notes on the Cornelian Cherry.* By Dr. X. Landerer.

The *Cornus mascula* grows plentifully in the gardens of Greece, where it is also indigenous.

Its fruit, called *krania*, is greatly prized by the Turks on account of its very agreeable acid flavor. They use the juice of it in different ices and sweatmeats, also to prepare their sherbets or *bosas*, which are very refreshing and very wholesome acidulated but sweet drinks. The fruit is also considered very styptic; and hence during the prevalence of cholera, it is the only fruit allowed to be sold in the streets and bazaars.

The syrup of *krania* is employed in Greece as that of the raspberry or blackberry is in Central Europe. Before the fruit ripens, it is put into vinegar, to preserve it all the year round, and in this state it is eaten like olives, mad-apples, and tomatoes.

This plant was well known to the ancients. Homer tells how the famous poisoner Circe gave its fruits to the companions of Ulysses to put them to sleep. Pliny relates that the fruit of this tree is useful against diarrhœa.

In the East they make use of the flowers also in cases of diarrhœa and hemorrhages, and the poor collect the kernels to make ornaments.—(*Dublin Hospital Gazette*, and *Pharmaceutical Journal and Transactions*, February, 1860.)

III.—*On the External Use of Cyanide of Potassium in Neuralgia, with Researches on its Physiological Action.* By M. TH. ROCHE, of Besançon. (Bullet. de la Soc. Méd. de Besançon, No. 8, p. 3, Besançon, 1859.)

The solution of two to three decigrammes of cyanide of potassium in thirty grammes of water cures neuralgia, if it is *superficial and localized.* This is the conclusion at which the author of this excellent essay arrives, and which he founds upon the observation of eleven cases, five of which only he reports in detail. In these eleven cases, which include ten of cephalic neuralgia and one of pleurodynia, nine cures were obtained, seven times by the simple and sole application of the topical remedy, and twice by associating it with the internal use of preparations of belladonna. In the two unsuccessful cases the treatment consisted of the external application of the cyanide and the internal administration of the narcotic solanaceæ. On comparing his own experiments with those of Professor Trousseau, the author arrives at the conclusion that the solution of cyanide of potassium, applied locally, does neither act by refrigeration, nor by rubefaction, vesication, or pulmonary absorption of the cyanic vapors, but by cutaneous absorption of the salt in the state of cyanide or free hydrocyanic acid. If this conclusion, deduced from therapeutical and physiological actions, cannot be supported by chemical demonstration, the reason must be looked for: (1) In the fugacity of the cyanic principles, particularly if they are in small quantity, and elaborated by their introduction into an organic apparatus; and (2) in the impossibility of experimenting on man with quantities sufficiently large to prove chemically the presence of a notable quantity of the absorbed principle.—(*Echo Médical Suisse*, and *Journal de Médecine de Bordeaux*, February, 1860.)

IV.—*On the Iodide of the Chloride of Mercury in the Treatment of Skin Diseases, and especially of Couperose and Acne.* By M. AL. DEVERGIE.

This preparation, which possesses very powerful properties, was introduced by M. Boutigny, about fifteen years ago, for the treatment of couperose, acne, and other skin diseases which are very obstinate, and often irremediable by ordinary means. In these cases, it is looked upon by many as a specific; but its chemical composition and therapeutical effects have not been studied with sufficient accuracy. The action of iodine on protochloride of mercury was first investigated by Planche and Soubeiran, (*Journal de Pharmacie*, t. xii. p. 651;) the product which they obtained presenting powerful escharotic properties. By varying the proportions of iodine and of protochloride, however, different compounds may be formed; and chemists have now ascertained that the so-called iodide of the chloride of mercury is not a definite chemical substance, but is a mixture of the chlorides and iodides of mercury, and generally consists of the bichloride and biniodide of mercury along with the protochloride. Such appears to be the composition of the substance manufactured by M. Boutigny, who, however, keeps his process secret. M. Devergie recommends the process of M. Dannecy, as yielding a product similar to that of M. Boutigny. Equal equivalents of iodine and bichloride of mercury are employed, dissolved in alcohol, which retains a great part of the bichloride in solution, and the salt which separates (iodide of the chloride) in this case consists of a large proportion of biniodide, and a very small quantity of the bichloride, mixed with an excess of calomel. Prepared in this way, the iodide of the chloride, like that furnished by M. Boutigny, is much less violent in its action than either the biniodide or the bichloride alone would be in the same dose.

M. Boutigny employs his preparations externally in the form of an ointment, internally in the form of pills or syrup. The formula of the pommade de Boutigny is: Axunge, ℥j; iodide of the chloride of mercury, 12 grains—but more recently he has increased the proportion of the iodide of the chloride to 16 grains, and the ointment, of this strength, is now usually employed. The pills contain about one-fifth grain in four pills.

When applied to the skin, this ointment usually produces, after the second or third application, a feeling of heat and smarting, which lasts during most of the night if the pommade is used in the evening. Next day, if the pain has not been severe, the skin is merely reddened; but if the irritant action has been fully developed, the red surface is covered by an immense number of minute serous vesicles, which quickly dry up, leaving an epidermic crust. In most cases, the inflammation which is excited subsides rapidly, so that the application of lard or cold cream, for four or five days, allows the skin to return to its natural condition. Such are the usual results; but the effects, of course, vary with the strength of the preparation and the degree of sensibility of the skin—circumstances which must be attended to in the treatment of different cases. M. Devergie's mode of application is to spread the pommade, in very thin layers, uniformly over the skin, by means of gentle inunction with the point of the finger, for about one minute; this is repeated every twenty-four hours for two, three, rarely four days, and then stopped; the inflamed surface is next covered with lard or starch powder for three or four days, till the excited action subsides, and the application of the ointment is then renewed as before. This treatment is continued for five, six, or eight months, or even one or two years in case of relapse. It is the general opinion, that to obtain a complete cure, the application must be repeated till the strong ointment, containing sixteen grains of the iodide of the chloride, ceases to exert any action on the skin; but M. Devergie considers the cure established when the skin is clear of eruption, and the ointment produces only a third or a fourth of its previous effects. It is especially in couperose, a disease always difficult, and sometimes impossible, to remedy by ordinary means, that the iodochloride ointment exhibits the most remarkable effects. When the disease has not reached the tuberculated stage, it frequently cures it without requiring so long as six or eight months of treatment. M. Devergie relates a striking example of this in the case of an actor, who, in consequence of intemperance, had his face so blotched and disfigured that even with the assistance of paint he could not venture to appear upon the stage, and had to give up his engagements. In a few months he was completely cured. Although the cases are not always so satisfactory, M. Devergie strongly recommends the use of the iodide of the chloride in pommade as generally a very successful means for the treatment of couperose. In acne, generally, it does not always suit. It succeeds least in A. sebacea and punctata; A. indurata yields most easily, and next to it A. miliaris. The acne rosacea does not usually require this treatment at all. In all these forms of acne, sulphureous baths and other measures are requisite. In mentagra and chronic lichen, it is not more successful than other analogous remedies; in lupus and eczema it does not appear to do any good. With regard to the mode of action of the iodide of the chloride, M. Devergie holds very decided views. He denies entirely its specific action, and maintains that it cures by its topical effects only, and not by any influence on the constitution. He strongly disapproves, therefore, of the internal use of the remedy as quite unnecessary, and as likely to give rise to salivation, and to injure the general health. According to his view, the cure is effected locally, by a process of substitution. The iodide of the chloride, being a powerful irritant, induces an acute inflammatory condition, which takes the place of, and, as it were, substitutes itself for, the chronic subinflammatory action of the disease; and by changing the mode of vitality of the tissue, promotes a return to the healthy state. The application of blisters in chronic inflammations of the skin is a common example of the same principle of treatment. Whatever may be the value of this theory, the general view is important, that it is merely in virtue of its stimulant or irritant properties, exerted locally, and not by any specific action, that the cure is accomplished. Finally, M. Devergie expresses the wish that in pharmacy some uniform method should be adopted of preparing the iodide of the chloride of mercury, so that it may be procured always of the same strength, and may be introduced into the therapeutics of legitimate medicine.—(*Bull. Gen. de Thérap.; Edinburgh Medical Journal*, December, 1859.)

### V.—*On Chlorine Lotions in Variola.* By Dr. Eisenmann.

Several young persons having died asphyxiated at Würzburg, in consequence of the development of pustules of variola or varioloid in the larynx, Dr. Eisenmann was induced to seek for a means which would limit the eruption to the skin, and prevent its propagation to the mucous membranes.

Most of the acute exanthemata take their origin in a mucous membrane, as scarlatina in that of the throat, and rubeola in that of the respiratory organs and eyes; and as long as it remains localized in the mucous membrane, and is moderate, the affection of the internal organs is not dangerous. But when the course of the subsequent eruption on the skin becomes impeded, or, that when it appears it is in such intensity or abundance that the economy does not possess sufficient energy to meet the assault, the primary affection of the mucous membrane may then so increase as to give rise to dangerous lesions. The indications which we should have in view are to favor the eruption of the exanthem, and prevent the skin becoming excessively overcharged with it. The asphyxia above alluded to took place when the eruption had undergone a vivid development, and had given rise to abundant pustules; and our object in such case should be to moderate the reflex action of the exanthem on the economy. The means of doing this, the author believes, will be found in applying over the whole surface, three or four times a day, weak, tepid, chlorined water, in imitation of a practice successfully pursued by M. Schönlein in scarlatina. He has now tried the plan during various epidemics, and has been able to confirm his own experience by that of others. The general conclusions are as follows:—

1. That chlorine lotions employed at the period of eruption present the following advantages: (1) They favor the development of the eruption, and thus mitigate febrile action. (2) The pustules are not too abundant, and do not become confluent. (3) There is no subsidence or repercussion of the pustules observed, nor any variolous affection of the mucous membrane or of an internal organ. (4) The patients suffer little during the height of the exanthem, preserve their appetite, and sleep well. (5) The course of the exanthem is very rapid, and there is neither suppuration, with its consecutive fever, or tumefaction, salivation, etc. (6) Scabs do not arise, only thin pellicles forming, which soon fall, without leaving any mark or cicatrix. (7) No consecutive affections are observed.

2. When resorted to only after the eruption has taken place, the lotions produce the following effects: (1) They diminish or disperse the inflammatory condition, and accelerate the course of the exanthem. (2) They prevent its repercussion and the propagation of the variolous affection to the mucous membranes and internal organs. (3) In cases in which the mucous membranes have already become affected, the lotions exert a derivative action; and if together with them gargarisms, chlorine inhalations, and chlorined water internally are had recourse to, the intensity of these complications is much diminished, so that recovery takes place in cases in which life seemed to have been in great danger. (4) Employed in good time, the lotions, even when the eruption has become developed, may yet prevent suppuration. If, however, this has taken place, it may still be moderated; and we find neither irritation of the skin, nor intoxication of the blood from absorption of pus, and consequently no general reaction. (5) Thin scabs only are formed, which soon fall off, leaving but temporary red marks. (6) No consecutive diseases arise.

This means is beyond comparison superior to the variety of ectrotic applications having for their object the prevention of pitting, for these, when they can be borne, at most confer this local advantage without diminishing the severity of the disease. It is true that applications of corrosive sublimate or tincture of iodine exert a somewhat similar action on the variola to chlorine; but no one would risk making repeated and general applications of the former; while the sudden arrest of the morbid process over a large surface by means of iodine, which also interrupts the functional action of the integument, would often prove anything but a safe procedure.—(*Bull. de Thérap.*, t. lvi. pp. 232-240, and *Med. Times and Gazette*, Feb. 11, 1860.)

VI.—*Pills of Carbonate of Ammonia in Chronic Bronchitis.* By DR. JOHN WILLIAMS, of Cork.

Dr. Williams recommends the following formula for administering carbonate of ammonia in pills, which, he says, are of great service in chronic bronchitis, and especially in those cases where the bronchial secretion is viscid and expectorated with difficulty:—

℞.—Ammoniaci, gr. ij;

Pulv. Ipecac. gr. $\frac{1}{2}$;

Morphiæ Mur. gr. $\frac{1}{6}$;

Ammoniæ Carb. gr. ij;

Mucilaginis Acaciæ, q. s.

Misce. Fiat pilula.

The pills should be at once coated with a varnish of balsam of Tolu, dissolved in chloroform, and they should afterwards be kept in a bottle.—(*Pharmaceutical Journal*, London, February, 1860.)

VII.—*Pencils of Tannin in Affections of the Uterus.* By DR. BECQUEREL.

This form of application, pointed out by Dr. Becquerel, seems likely to be of service in the treatment of lesions affecting the cavities of the neck and body of the uterus. In particular, in the fungous conditions of their mucous membranes, with consecutive hemorrhages, the tannin pencils might be advantageously substituted for the intra-uterine injections, which are not always free from danger. Dr. Becquerel's formula is: ℞.—Tannin, 4 parts; gum tragacanth, 1 part; bread-crumb, q. s. to give the proper consistence. These pencils are five millimetres in diameter, and three centimetres long. To use them, the neck of the uterus is exposed by means of the speculum; a pencil of tannin is introduced with the forceps into the os tincæ, and is then pushed into the uterine cavity, and secured there by a plug of lint soaked with concentrated solution of tannin. Once in position, the pencil softens and dissolves, and modifies the tissues with which it is in contact. At the end of twelve hours, the plug of lint is withdrawn by means of a thread attached to it. Every three or four days a new pencil is introduced in the same manner; and after a month of this treatment, the fungous state of the mucous membrane progressively disappears, and the hemorrhages are arrested.—(*Bull. Gén. de Therap.*, and *Edinburgh Medical Journal*, January, 1860.)

VIII.—*Treatment of Burns by the Local Application of Distilled Cherry-Laurel Water.* By DR. E. FRANCHINO.

The use of cherry-laurel water in local applications is not a novelty; it has been employed in the treatment of erysipelas, neuralgia, ulcers, certain kinds of ophthalmia, (as collyrium,) hemorrhoides, rheumatic pains, etc. M. Franchino has used it in three cases of burns of the second, third, and fourth degree of Dupuytren, which it has cured with great promptness. Among other advantages, cherry-laurel water possesses that of suppressing the pain almost completely, of calming the agitation, the heat, etc. M. Franchino mixes it, in proportion of eight parts to a hundred, with solution of gum arabic, and applies compresses, soaked in this mixture, upon the burnt surface after having it previously cleaned, and having pierced the phlyctenæ. In order to renew the dressing the compresses must be softened, before their removal, by covering them with other compresses soaked in water.—(*Gazetta Medica Italiana, Stati Sardi*, No. 37, and *Gazette Hebdomadaire*, February 24th, 1860.)

IX.—*Treatment of Excessive Perspiration of the Feet.* By M. GAFFARD.

It is well known that excessive perspiration of the feet may be a very troublesome complaint. It not unfrequently produces excoriation between the toes, giving rise to an exudation of a disgustingly fetid odor; and it sometimes occasions ulcerations, which render locomotion very painful, or altogether impossible, forcing the persons affected to interrupt their business occupations. M. Gaffard, of Aurillac, recommends the following means, which he says he has employed in such cases with complete effect. The treatment consists in pouring between the toes a few drops of a liquid, composed of one gramme (fifteen grains) of red oxide of lead, and twenty-nine grammes (about an ounce) of the solution of subacetate of lead, (of the French Pharmacopœia;) the sesquioxide of lead is pounded in a mortar of porcelain till it is finely divided; the subacetate is added gradually; and the whole is put in a bottle, which is shaken each time it is used. This application made every eight days is sufficient, in most cases, according to M. Gaffard, to cure the affection, and prevent its return. The liquid, without completely stopping the perspiration, moderates its amount, and regularizes the action of the morbid surfaces. The perspiration becomes inodorous, the skin regains its original thickness at the excoriated parts without losing its pliancy, and the parts return to the natural condition of cleanliness and health.—(*Rép. de Pharmacie*, and *Edinburgh Medical Journal*, January, 1860.)

X.—*Treatment of Ascarides.* By M. HERVIEUX.

M. Hervieux, after relating an interesting case in which the irritation produced by ascarides in an adult was long mistaken for severe rectitis, communicates the results of his experience in the treatment of this troublesome affection in children. In the first place, of all purgatives castor-oil is the best; and this statement is made after a full trial of salines, calomel, scammony, jalap, etc. The oil always gives rise to a greater or less evacuation of the worms, giving the patient a truce of some months. It is true that it is only a palliative. But where is an agent for a radical cure to be found? Mercurial ointment, passed in upon the finger, is of no durable efficacy; and the daily injection of cold water does not destroy the disease, but acts only as a palliative. Still, this last is the best means to adopt when castor-oil cannot be taken. Injections of olive oil, of sulphuret of potassium, of lime-water, or of corrosive sublimate, which have been prescribed by practitioners in cases which have since come under M. Hervieux's notice, have never led to a definite cure. Time seems, indeed, to be the sole agent capable of completely destroying these helmintha; and M. Hervieux has met with numerous individuals who during childhood had suffered much from ascarides, and who, having reached the adult age, no longer gave any signs of them. In a few instances, however, the worms are still met with at an advanced period of life. The female worm is distinguished from the male, among other marks, by being nearly twice as long, and in the numerous cases in which he has searched for her, M. Hervieux has never yet been able to detect any females amid thousands of worms.—(*Bull. de Thérap.*, t. lvi., and *Medical Times and Gazette*, February 11, 1860.)

XI.—*Infusion of Coffee in Strangulated Hernia.* By M. RONZIER JOLY.

M. Ronzier Joly, of Clermont, relates two additional cases in proof of the utility of the practice, imported from Havana, of administering repeated doses of strong coffee in the case of strangulated hernia. In both these cases of inguinal hernia the success of the coffee does not seem to have been at all doubtful, although the association with it of frictions of belladonna made over the hernial tumor may have aided in producing the favorable result. In both cases, however, an after consequence was met with, which had not occurred to the author before, although he lives in a district in which hernia is of very frequent occurrence. This consisted in a true hemorrhagic or dysenteric flux, for the relief of which large doses of bismuth proved of great utility. The production of this flux he attributes to the coffee, indicating that some caution is required in its administration.—(*Ibid.*)

XII.—*Muriate of Ammonia in Nervous Cephalalgia.* By PROF. BARALLIER.

Professor Barallier, of Toulon, reports that within the last three years he has administered this substance in 259 cases of nervous cephalalgia, and with success in 202 of these. He gives forty-five grains, combined with mint-water and syrup of orange-peel, divided into three doses, to be taken at intervals of half an hour, amendment commencing after the first dose, and the third frequently not requiring to be taken. To prove effectual, however, the remedy should not be given at the very commencement of a paroxysm, but when it has acquired great intensity. This agent not only gives relief to the urgent pain of the paroxysms, but, after having been had recourse to on several occasions, diminishes the number and frequency of these. To be of use, it must not be indiscriminately used for every cephalalgia; and the result of the analysis of M. Barallier's experience leads to the following conclusions: 1. The muriate almost constantly dissipates paroxysms of idiopathic migraine, and of migraine consecutive to too abundant menstruation. 2. It is powerless in the hemicrania which is dependent upon irregularity or suppression of the menses. 3. It is tolerably successful in cranial pains dependent upon disorder of the stomach, and in the accidental cephalalgia frequent in women and feeble persons under the influence of sudden changes of the atmosphere, prolonged intellectual labor, or moral emotion. 4. It operates beneficially in cephalalgias consecutive to repeated paroxysms of intermittent fever; those which are observed during the decline of severe fever, and in the course of the irritative period of typhus.—(*Ibid.*)

XIII.—*A New Method of Preparing and Applying Chloride of Zinc.* By G. W. SPENCE, M.D.

Dissolve fifty grains of prepared chalk in two drachms (by measure) of commercial muriatic acid; dissolve a hundred and fifty grains of sulphate of zinc in two fluidrachms of boiling water. When required for use, mix the two solutions, and the result will be a paste weighing nearly an ounce, and containing about one-sixth of pure chloride of zinc. The proportions are nearly, but not exactly, those indicated by the atomic weights. A little study would easily produce a paste of harder or softer consistence.—(*Lancet*, and *British Am. Jour.*, February, 1860.)

XIV.—*Natural History, Properties, and Medical Uses of the Sanguinaria Canadensis.* By DR. GIBB. (Read before the Medical Society of London.)

The author went most fully into the early history and botanical characters of the plant, the first notice of which was by Jacob Cornuti, in 1635. It was cultivated in England prior to 1640. He showed the metamorphosis of denomination it had undergone till the time of Linnæus, who settled the name of the plant. It belongs to the sexual system Polyandria Monogynia, and the natural order Papaveraceæ, and is common to a large portion of the North American continent, especially Canada, where it was first discovered. It is perennial, and the only officinal part is the rhizome, which abounds in an orange-red colored juice. As met with in commerce, it is in the form of dried root, and, from its cheapness, is unadulterated. The box of specimens exhibited was from Messrs. Lyman, Savage & Co., of Montreal, and contained eight pounds. The odor of the root is somewhat narcotic, and, on handling, causes sneezing. The qualitative analysis, as determined by the author and other observers, is as follows: 1, sanguinarina; 2, porphyroxin; 3, puccine; 4, chelidonic acid; 5, fecula; 6, saccharine matter; 7, vegetable albumen; 8, orange-colored resin; 9, fixed oil; 10, extractive matter; 11, lignin; 12, gum, (a little.) The sanguinarina is an alkaloid, discovered by Dr. Dana, and contains the active principle of the plant. The porphyroxin was extracted from it by Riegel, and is analogous to the same principle discovered by Merck in opium. Puccine is a third principle, discovered by Mr. Wayne, of Cincinnati; and this name was given to it by the author, after the Indian appellation of the plant. Several of the other ingredients were detected by the author himself.

A series of experiments were performed on man and animals, by the author, and Dr. Fenwick, of Montreal. They went to show that, in its concentrated form, bloodroot was extremely irritating to man and animals, affecting principally the mucous membrane of the stomach and bowels. An excessive quantity acts as a poison, and produces violent vomiting, a burning sensation in the stomach, tormenting thirst, faintness, vertigo, indistinct vision, and alarming prostration of strength. Its properties, in regulated doses, are those of an emetic: nauseant, expectorant, and diaphoretic. A narcotic, sedative, stimulant, and alterative property is occasionally exerted. As an emmenagogue, it has long been known; and it is used as an escharotic and errhine. As an emetic and expectorant, it is highly valuable in various chest and throat affections; and has been employed in pneumonia, phthisis, bronchitis, catarrh, asthma, croup, diphtheria, cynanche maligna, and pertussis. As a diaphoretic, stimulant, and alterative, it is administered in many diseases in which sudorifics are indicated. In scarlatina, rheumatism, jaundice, dyspepsia, hydrothorax, and some other affections, its virtues have been praised by many practitioners. The general experience of those who have tried it in cancer is, that it is perfectly inert in that disease; and it is satisfactorily proved that it was not originally employed by the Indians of the shores of Lake Superior to cure cancerous affections. Its value, locally, in many skin affections, is undoubted, and it is certain to cure many obstinate forms of head eruptions.

Several American physicians have given their testimony to its value in some of the stages of pneumonia, and especially in the chronic form. As an expectorant in the first and second stages of phthisis, its action is said to be very reliable. The author found it especially serviceable in the pretubercular, the second and third stages of the disease, and not less valuable in bronchitis. Short abstracts of several cases of phthisis in different stages were related, to illustrate the good effects of the remedy; in all of which the expectoration became easy, the breathing clearer, the spasmodic efforts at coughing less, and even in the last stage much improvement resulted for a time. As an expectorant and mild stimulant in the second and third stages it cannot be surpassed, and materially helps to prolong life even in very hopeless cases. In consumption, associated with disease of the windpipe and throat, the tincture is useful in promoting warmth and easy expectoration. In chronic bronchitis it is in most general use in North America, as one of the most active and useful expectorants; and the author has for several years found it more serviceable in this disease than many other remedies. It will allay the cough and irritation in some forms of follicular inflammation of the throat, associated with phthisis or bronchitis. Not less serviceable is it in various forms of catarrh, particularly in the chronic, associated with emphysema. In coryza, or cold in the head, it is much employed, and has been found useful by the author. The paroxysms of asthma are relieved by it, and their severity and frequency diminished. It is a remedy in common use for pertussis in the United States; but although it had cured cases under the author's care, he did not recommend it as superior to other remedies. It is also much employed in croup, in the membranous form. As an emetic in the croupal form of diphtheria, it acts with energy, and produces a thrilling effect upon the entire mucous membrane of the fauces and respiratory tract, with a feeling of warmth. It alone seems to impart vitality to the suffering throat, and is recommended by the author with confidence, in the form of decoction or infusion, four to eight drachms, at short intervals, until vomiting ensues; it is then to be followed by steel and other preparations recommended for this disease. In the malignant form of diphtheria, besides active and energetic treatment, an acetous decoction of bloodroot as a gargle will prove invaluable. Its usefulness in epidemic malignant scarlatina has been fully tested by Dr. Jennings, of Virginia, in the same form of gargle; a fact which should be borne in mind when this epidemic appears. Some evidence was also afforded of its good effects in certain forms of chronic rheumatism, and in some hepatic affections. In amenorrhœa, it will prove, either alone or combined with other substances, one of the best emmenagogues. The skin diseases which have been cured by it in the form of ointment are scabies, tinea capitis, impetigo of the scalp, and many others. The preparations in use are—the powder; the compound powder of the author; the powder with camphor; the infusion, decoction preserved juice, and oil; the extract, tincture, wine, vinegar, syrup, and ointment.—(*Lancet*, Am. ed., April, 1860.)

XV.—*Inhalation of Chloroform.* By Dr. BERAUD.

An interesting paper has just been communicated to the French Academy of Medicine by Dr. Beraud, on the subject of Dr. Faure's method of administering chloroform. This method consists in causing that agent to be inhaled by one nostril only, the other remaining, meanwhile, in free communication with atmospheric air. The apparatus is extremely simple, consisting of a bottle with two necks or tubulatures, and capable of containing one hundred grammes of water. An india-rubber tube with a tapering end is adapted to one of the necks, and is intended for insertion into the nostril; the other neck remains open, the operator stopping it with his thumb when necessary. The tube is seventeen centimetres in length, and has a diameter of at least thirteen millimetres. To use this apparatus, pour about ten or twelve grammes of chloroform into the bottle, and having stopped the open tubulature with your thumb, let the patient receive the tube into his nostril, recommending him to breathe naturally. There being no communication between the bottle and the atmosphere the chloroform does not evaporate, and the patient is not aware of any unpleasant sensation.

The subject having now acquired the habit of breathing in that way, the operator gradually slackens the pressure of his thumb, and allows a little air to enter, by which means the patient inhales atmospheric air charged with a little chloroform. From that moment, according as the painful sensation increases or diminishes, the outer air is alternately admitted or excluded, until the thumb being entirely withdrawn, the patient receives the full quantity required. The operation may also be conducted thus: Let the patient breathe through the empty bottle, and then introduce a drop of chloroform, then another, and so on gradually. The great point is not to allow the effluvia of chloroform suddenly to exercise too irritating an influence upon the respiratory organs. After the second or third minute the operator should shake the bottle, so as to project the liquor on its sides, by which the evaporating surface is considerably increased. Should the patient happen to open his mouth, the operator must close it with his hand. By this process the patient feels no pain, no sensation of suffocation or dyspnœa, nor is there any congestion of the brain. The state of anæsthesia may be continued with the greatest ease, and without danger, by keeping the tube ready to be again introduced into the nostril, if necessary; nor is there any possibility of sudden asphyxia, as the effects of the agent develop themselves very gradually.—(*Times*, and *Pharmaceutical Journal and Transactions*, February, 1860.)

XVI.—*Remarks on Propylamin.* By DR. H. STABLER, of Alexandria.

In a letter addressed to the editor of the *American Journal of Pharmacy* we find the following remarks on propylamin, which was so highly recommended by

Dr. Awenarius, of St. Petersburg, as a remedy in acute and chronic rheumatism, (see this journal for January, 1859, p. 180,) and for the preparation of which a formula was published by Professor Wm. Procter, in the *American Journal of Pharmacy* for March, 1859:—

"I prepared a portion of propylamin from herring pickle, soon after reading the article, and induced some of our physicians to use it in their practice, which resulted in establishing its reputation among us as an indispensable remedy. It has been in use here about nine months, and has been administered in about fifty cases with most marked effect, seldom failing to give relief in twenty-four or thirty-six hours in acute cases, and a longer time in chronic rheumatism. I think its great value is demonstrated in acute rheumatism of young subjects, where the disease, unless quickly controlled, is so liable to attack the structures of the heart. One case of the kind presented in the practice of Dr. I. J. Murphy, of this place, in a little boy: commencing with swelling of the knees, ankles, and other joints, high fever, full, bounding pulse, and other symptoms promising a long, tedious case under the usual modes of treatment; he was directed to take the propylamin in the usual doses, (two or three drops in water for an adult,) and was convalescent in three days. Another physician told me, a few days ago, he had used it with much satisfaction and uniform success.

"A good deal has been published on the preparation and administration of the remedy in the *Journal* and other periodicals, but nothing as to its use in this country that I have met with; thinking it might interest thy readers to describe our experience with it, and perhaps induce further trials of it, is my apology for troubling thee with this letter."—(*American Journal of Pharmacy*, March, 1860.)

XVII.—*On the Use of the Hypophosphites of Soda and of Lime in the Treatment of Phthisis.* By RICHARD QUAIN, M.D.

In this article Dr. Richard Quain states his experience in regard to the use of the hypophosphites of soda and lime in the treatment of phthisis, as recommended by Dr. J. Francis Churchill, of Paris, in a communication read before the French Academy of Medicine in July, 1857. After reviewing Dr. Churchill's theory on the pathology of phthisis, and on the mode of action of the hypophosphites in this disease, the author continues as follows:—

"Encouraged by statements like this, and by a lengthened catalogue of the phenomena of improved health, which it was said resulted from the use of those remedies in Dr. Churchill's hands, I determined on giving them a fair trial in a certain number of cases. They were, therefore, administered in twenty-two cases, taken, without selection, from among the ordinary in-patients of the Hospital for Consumption, at Brompton. Of this number (twenty-two) twelve were males, and ten females.

"*The stage of the disease.*—Two cases were in the first, ten in the second, and ten in the third stage of phthisis.

"*The dose of the remedy.*—Dr. Churchill recommends ten to thirty grains as the dose, of either the hypophosphite of soda or of lime, daily, in a simple fluid. The dose to be increased until the general symptoms disappear. In some cases he prefers the one salt to the other. For example, he thinks that the salt of lime checks the expectoration, and thereby increases the cough; while the salt of soda is less energetic in its action. I met with nothing confirmatory of this impression. The dose given to the patients at Brompton was, in the first instance, ten grains, three times a day, except in the case of a child, where only five were given. The disease progressing, or being stationary, or the effects of the remedy being *nil*, the dose was gradually increased. Thus, in four cases, it was increased to a drachm three times a day; in ten cases, the dose reached two scruples or more; in eight, the dose remained under half a drachm. It will thus be seen that the remedy was given freely. In no case, let me add, was there any appearance of the troublesome symptoms indicated by Dr. Churchill as following large doses.

"*The duration of the treatment.*—One case was under treatment for six months, one for four months, six for three months, nine for two months, five for one month. During this lengthened course of treatment, I looked anxiously, but in vain, for those marked physiological effects described by Dr. Churchill. There were no evidences of the "improved powers of innervation;" "the hair and nails did not grow more rapidly;" there was no "appearance of plethora or of fullness;" the patients did not describe "an unaccustomed sensation of feeling better and stronger after a few doses of the remedy." Nay, I would say that there was nothing more felt by the patient, nor noticeable by the physician, than if so many grains of carbonate of soda or prepared chalk had been taken.

"*The results.*—To return, then, to the more immediate object for which these agents were administered, viz., to ascertain their value in the cure of consumption, I have to state, that of the twenty-two cases, six were more or less improved while under treatment. Of these six, three were improved in but a slight degree, and only for a short time; in three the improvement was marked, but in one only of the latter has the improvement been permanent; of the two other cases, one continued using the hypophosphite for three months after leaving the hospital, during which time she grew gradually weaker, and finally died; the other, a man, after leaving the hospital, continued the treatment for some time, but gradually grew worse, and is now dying. All the other sixteen cases steadily lost ground while using the hypophosphites in the hospital. Happily, in six of these cases, the treatment by hypophosphites was suspended, and the usual treatment by cod-liver oil, tonics, etc., being substituted, a decided improvement in each was the result."

In order to illustrate the chief features of the treatment by the hypophosphites, the author reports the history of seven cases, including the three in which alone any useful result *seemed* to follow the treatment. At the end of his communication he states the *conclusions* at which he has arrived, in the following words:—

"Reviewing the cases of which the preceding may be said to be types, we see that of twenty-two individuals laboring under phthisis, submitted to the hypophosphite treatment, sixteen derived no benefit whatever; in three the benefit was so slight and temporary as scarcely to deserve notice; in two the improvement, though marked, was temporary; and in one case the result has been satisfactory and permanent. Small as the therapeutical powers of the hypophosphites are shown to be by these facts, are we justified in assigning to them even thus much? I think not. For we cannot forget that our cases are hospital cases; that, oppressed by sickness, care, and anxiety, they come from close, unhealthy localities; that they were more or less destitute of good food and good air. When they enter the hospital, they begin to feel the influence of hope; they live in warm, airy, and well-ventilated wards, find agreeable occupations, and have plenty of good food. Under such circumstances, the patients frequently improve in health, without the application of any medicinal agents. It would therefore be as fair to attribute the slight or temporary improvement which took place in some of these cases to hygienic as to the therapeutical agencies. Nay, further, this opinion is confirmed by the fact that two or three cases which did best in the hospital ceased to do well when they left it.

"Desirous of otherwise testing the value of these substances, I thought it would be well to compare the results of my ordinary hospital practice with that of the hypophospite treatment. With this view, I requested my friend and late clinical assistant, Dr. Hill, (to whom I am indebted for much assistance in this inquiry, and for the notes of the preceding cases,) to make abstracts of any twenty-two successive cases in the hospital books. He did so, and having ascertained the results of the treatment in these cases, I find that he has given me notes of eleven males and eleven females, of whom three were in the first stage of the disease, five were in the second, and fourteen in the third. It will be remembered that twelve were in the first and second stage, and ten in the third. Thus in the former cases, the advantage was in favor of the hypophosphite cases, so far as the stages of the disease were concerned; nevertheless, we find that of the cases submitted to other treatment, sixteen were more or less materially or permanently benefited, while in six only did the disease progress unfavorably. Exactly the converse was the case when the hypophosphites were given. Thus there were sixteen of twenty-two cases unrelieved. This comparative evidence is further strengthened by bearing in mind that six of the cases in the former series, which were unrelieved by hypophosphite treatment, did well subsequently under other treatment.

"A review of the preceding facts has led me to form a most unfavorable opinion of the value of hypophosphites in the treatment of phthisis. I believe them to be comparatively, if not absolutely, useless. I have been induced to take some little pains in investigating the subject, because of the unhesitating confidence with which their value is asserted and their use recommended in certain quarters, and I have also seen in the cases of some patients who have visited Paris, how much time has been thrown away by substituting the use of these salts for remedies of undoubted efficacy in controlling the progress of phthisis."—(*London Lancet*, March 17th, 1860.)

### XVIII.—*Saccharated Lime.*

Lime dissolves in water in much larger proportion in presence of sugar, and this solution is strongly recommended as a tonic and antacid by Dr. John Cleland.

Dr. Cleland gives the following formula for its preparation:—

Slake eight ounces of quicklime; rub up with it five ounces of white sugar; add one pint of water; stir for some time, till the hard, stiff masses which the sugar and lime are liable to run into are as much as possible dissolved; then filter. The product should be perfectly clear, and of only a slightly yellowish tint. It should be kept in bottles, well stoppered. Dose, 20 to 60 minims, in water, after meals.

This preparation, Dr. Cleland asserts, does not weaken digestion like the alkalies, but on the contrary, is an important tonic in dyspepsia. While it corrects diarrhœa dependent upon disordered digestion, it is a valuable means of overcoming the chronic constipation owing to dyspepsia. It may be found useful also in allaying the cravings of the intemperate.—(*Edinburgh Med. Journal*, August, 1859.)

XIX.—*On the Seat of the Vesicating Principle of Lytta Vittata.* By PROF. JOSEPH LEIDY.

Professor Leidy has satisfied himself, by a series of well-devised experiments, that the vesicating principle of this fly belongs to the blood, the peculiar fatty substance of certain accessory glands of the generative apparatus, and to the eggs.

The active principle of cantharides was thought by Pereira and others to reside in the generative organs of the animals.—(*Am. Journ. Med. Sci.*, January, 1860.)

---

### TOXICOLOGY AND LEGAL MEDICINE.

I.—*Toxicological Remarks on Nitro-Benzine.* By DR. CASPER.

Nitro-benzine or nitro-benzide, discovered by Mitscherlich in 1834, is obtained by treating benzine with fuming nitric acid; it is a liquid substance at the ordinary temperature, yellow, of sweet and pleasant taste, and exhaling a strong odor of bitter almonds. It crystallizes at + 3 degrees, and dissolves easily in alcohol, ether, and the oils, very little in water. Its composition is represented by the formula, $C^{12}H^5$ $CezO^4$.

This substance is very generally applied in perfumes, as a substitute for the essence of bitter almonds and hydrocyanic acid; the druggists sell it in large quantity, and as its price is not very high, there is reason to fear that sooner or later it may give rise to cases of accidental or criminal poisoning. Dr. Casper has ascertained by experiments on the rabbit and dog, that it possesses very energetic poisonous properties.

The blood and the different organs of animals poisoned by nitro-benzine emit an intense odor of bitter almonds. But as most toxicologists of the present day assume that this odor is sufficient to characterize poisoning by prussic acid, it is important to be aware of the fact, that in a case of poisoning by nitro-benzine the same characteristic circumstance is observed. Further investigations will, no doubt, serve to establish differential marks. M. Casper has noticed, in this respect, that the odor of nitro-benzine persisted, in his experiments, for several days after death, while hydrocyanic acid commences generally to decompose on the second day. It will be useful to bear this difference in mind.—(*Vierteljahresschrift für gerichtliche and öffentliche Medizin*, t. xvi. p. 1, and *Gazette Hebdomadaire*, February 24, 1860.)

II.—*On the Detection of Phosphorus.* By DR. FRED. HOFFMAN.

The author has instituted a series of more than one hundred and fifty experiments with Mitscherlich's method for detecting phosphorus. He has mixed the phosphoric mass of matches in different preparations, with food cooked in various ways, with beverages commonly used, and with medicines in a liquid, soft, and plastic state, and kept the mixture for a variable length of time. The presence of phosphorus, it will be remembered, is established by the phosporescence imparted to the aqueous vapors on distilling the liquid or liquefied mixture, and the distillation of the phosphorus is increased by the addition of sulphuric acid, chloride of sodium, sugar, etc., whereby the boiling point is made to approach that of phosphorus.

In the place of Mitscherlich's apparatus, the author has constructed a more simple one, which is easily prepared and readily taken apart. It consists of an ordinary flask, connected with a receiving bottle by means of a glass tube, which passes about eighteen inches through a glass cylinder, filled with cold water. A long, straight tube conducts the gaseous products from the bottle. The lamp and flask are surrounded with dark paper, and about three-fourths of the glass cylinder. The operation is best performed in a dark room.

The phosphorescence of the liquid increases in intensity with the consistence of the liquid and the quantity of the phosphorus. The gas bubbles are luminous, rise in the mixture, and apparently burn upon its surface with a bright flame. With the temperature the light is increased; a photosphere fills the flask, rises in the tube, and moves up and down within the cooled part. Sometimes only a column or a luminous ring appears stationary at the point where the vapors are cooled, and a luminous fog or sparks gradually sink into the receiver, or a sudden, frequently repeated lightning is observed. If the heat is raised too high, or the cooling is insufficient, the luminescence passes through the long gas-tube, at the mouth of which the gases take fire, if the volatile oils from cruciferæ (mustard, etc.) have been present.

Coffee, mustard, smoked meat, highly-seasoned food and beverages, and medicines containing odorous gum-resins, volatile oils, musk, castor, camphor, chlorine, etc., have the property of covering the odor of a small portion of phosphorus; minute quantities, which yet produce the symptoms of gastro-enteritis, may not be recognized by the odor in the contents of the stomach. The reaction is not prevented, except by the presence of much alcohol, volatile oils, and mustard. If the quantity of phosphorus be not too insignificant, the phosphorescence is observed, either momentarily or constant, at the beginning of the distillation, and after the alcohol has passed over.

The reaction is not interfered with by the presence of ipecacuanha, tartar emetic, magnesia, hydrated oxide of iron, musk, castor, opium, albumen, neutral, acid, or basic salts and double salts, volatile organic acids, chlorides, iodides, and sulphides, and by free acids; but iodine, chloride and bichloride of mercury in considerable proportion, and metallic sulphides in the presence of free sulphuric acid, and particularly oleum cinæ, (artemisiæ,) interfere with or prevent the reaction.

Numerous experiments, by distilling the brain of various animals, blood, albumen, casein, fibrin, legumin, and other protein compounds with diluted sulphuric acid, yielded not the least photospheric reaction.—(*Archiv. d. Pharm.*, Oct. 1859, and *Am. Journ. of Pharmacy*, March, 1860.)

III.—*Arsenical Fly-Papers.* By R. H. Brett, Ph. D., M.R.C.S., etc.

These papers differ considerably in weight, the lighter weighing about fifty grains, the heavier from eighty-five to ninety grains. They were all well dried before weighing. These papers may be divided into two classes: the one, "Papier Moure," containing the arsenic in the form of arsenic acid combined with potash; the other, arsenious acid, not combined with a base. Both sorts, when burned, gave out the peculiar odor of metallic arsenic; but the papers called "Papier Moure," yield an ash having a strong alkaline reaction, from which ash water dissolves carbonate of potash, readily detected by the ordinary tests; the other class of papers do not furnish any potash by burning. When the "Papier Moure" are boiled with distilled water, a solution is obtained, which is precipitated white by a salt of lime, brick-red by a neutral solution of nitrate of silver, and yellow by hypo-sulphite of soda and muriatic acid when heated, indicating the presence of arsenic acid. Both sorts of paper, when boiled with diluted muriatic acid and bright copper-wire, deposit metallic arsenic upon the copper surface, which deposit, when heated in a test-tube, furnishes a crystalline sublimate of arsenious acid. The metallic arsenic is, however, more readily thrown down from the papers where the arsenic exists in the form of uncombined arsenious acid than from the other description; the experiment, nevertheless, is sufficiently striking in either case. The quantity of arsenic in these several papers, estimated as *arsenious acid*, does not materially differ, with the exception of one of the arsenious acid papers, where nearly double the amount was obtained; indeed, it was found that when only about two square inches of this paper were boiled with fine copper wire and diluted muriatic acid, so much metallic arsenic was separated that the latter came off the copper surface, by simple agitation in water, in the form of small black flakes. As the "Papier Moure" gives a bitter-sweet extract to alcohol, it was examined for strychnine; this alkaloid, however, could not be detected. There is reason to believe that the bitter taste is due to quassia mixed with a little saccharine matter. The average weight of the fly-papers operated upon may be taken at about sixty-nine grains, and the average percentage of arsenic, estimated as *arsenious acid*, (not including the paper yielding fifteen per cent.) at about 7·69 grains, thus giving to each paper as much as 5·3 grains of arsenious acid. Two or three of these papers contain thus sufficient arsenic to poison a whole family.—(*Pharmaceutical Journal and Transactions*, February, 1860.)

IV.—*Arsenical Poisoning by Paper.*

1. Three children, near Tipton, have suffered from the arsenical emanations from a green bed-room paper in a newly-papered house. The symptoms were emaciation, pining, general restlessness, (worse at night,) and twitching of the facial muscles. Dr. Ballenden, observing these symptoms, concluded that they were suffering from the effects of gradual poisoning; and, on being removed into another room, the children recovered.—(*London Lancet*, February, 1860.)

2. Mr. R. Biggs, of Bath, reports the case of a watchmaker, who consulted him about a very troublesome and irritable condition of the mucous membrane of the mouth, from which he had been suffering for some time. On examination, Mr. Biggs discovered several small superficial ulcers extending along the inner surface of each lip, varying in size, the smallest being mere specks, the largest about one-twelfth of an inch in diameter. The smarting and annoyance were intolerable, and much increased in the evening, when the lips swelled and became so painful that eating, or even speaking, could hardly be indulged in; together with these symptoms there was profuse ptyalism. Different modes of treatment were pursued without producing any, or only a temporary improvement. Happening one evening to go into the patient's shop, the author perceived, on entering, an unpleasant smell, which, on inquiry, he found was produced whenever the patient lit the gas-jet by which he worked, as the heat caused a smell from the varnish covering his paper shade. The author's attention having thus been drawn to the shade in question, which was of a bright-green color, he applied Reinsch's test, and obtained immediate proof of the presence of arsenic, (aceto-arsenite of copper.) All treatment was left off; the patient discontinued the use of the shade, and complete recovery ensued, though not till after the lapse of nearly three weeks.

This case, remarks the author, is interesting in connection with the subject of arsenical paper-hangings, about which there has been so much excitement. It is certain that, while much truth was elicited in the controversy, there was a good deal of error superadded. Several articles were published to prove that the arsenic contained in the paper was volatilized by the heat of the gas in a room, the authors not remembering or not knowing that a temperature considerably above that of boiling water was necessary to produce this result. The rational conclusion finally arrived at was, that the symptoms suffered by the patients were caused by the mechanical separation of the green color, and its subsequent inhalation or absorption. In this case, however, was shown the effects of its actual volatilization, and the short time necessary to produce serious symptoms. The patient had used the shade but a few days before he began to suffer, and the period in which he so much improved was an interval during which he had not worked by gas light; but it should be observed that the temperature of the shade was elevated to a very high degreee, and that, when working, his mouth and nostrils were two inches distant from it.—(*Lancet*, and *Dublin Medical Press*, January 18, 1860.)

V.—*Detection of Blood Stains.* By M. BRUCKE.

In medico-legal inquiries, it is often of the utmost importance to determine the character of red spots on linen or steel, supposed to be blood stains. M. Brucke has recently published the following method, as being superior to those in common use:—

"Wash the spot with cold distilled water. To the reddish liquor thus obtained add a solution of sea salt, and evaporate to dryness, in vacuo, over a vessel containing sulphuric acid. Examine the dry residue well through a microscope, in order to verify whether it contains any matter that might be mistaken for Tetchmann's crystals; then add a little highly-concentrated acetic acid; evaporate again to dryness; moisten the residue with water; and then, if there really be blood in the spots, the microscope will reveal unmistakable crystals of hæmatin."—(*London Lancet*, January 14, 1860.)

